# Ordered arrays of nanoporous gold nanoparticles

**DOI:** 10.3762/bjnano.3.74

**Published:** 2012-09-13

**Authors:** Dong Wang, Ran Ji, Arne Albrecht, Peter Schaaf

**Affiliations:** 1 Chair Materials for Electronics, Institute of Materials Engineering and Institute of Micro- and Nanotechnologies MacroNano®, Ilmenau University of Technology, POB 10 05 65, 98684 Ilmenau, Germany; 2 SÜSS MicroTec Lithography GmbH, Schleissheimer Str. 90, 85748 Garching, Germany; 3 Center for Micro- and Nanotechnologies, Ilmenau University of Technology, POB 10 05 65, 98684 Ilmenau, Germany

**Keywords:** dealloying, dewetting, nanoimprint lithography, nanoparticles, nanoporous gold, ordered arrays

## Abstract

A combination of a “top-down” approach (substrate-conformal imprint lithography) and two “bottom-up” approaches (dewetting and dealloying) enables fabrication of perfectly ordered 2-dimensional arrays of nanoporous gold nanoparticles. The dewetting of Au/Ag bilayers on the periodically prepatterned substrates leads to the interdiffusion of Au and Ag and the formation of an array of Au–Ag alloy nanoparticles. The array of alloy nanoparticles is transformed into an array of nanoporous gold nanoparticles by a following dealloying step. Large areas of this new type of material arrangement can be realized with this technique. In addition, this technique allows for the control of particle size, particle spacing, and ligament size (or pore size) by varying the period of the structure, total metal layer thickness, and the thickness ratio of the as-deposited bilayers.

## Introduction

Metallic nanoparticle arrays are attracting more and more attention due to their potential applications in plasmonics [1–2], magnetic memories [3], DNA detection [4], and catalytic nanowire growth [5]. Nanoporous gold is very interesting for application in catalysis [6–7], for sensors [8], for actuators [9–10], and as electrodes for electrochemical supercapacitors [11]. This is due to the unique structural, mechanical and chemical properties of this material [7,12]. Nanoporous gold, already synthesized in the form of nanoparticles, possesses a much higher surface-to-volume ratio than bulk nanoporous gold films and gold nanoparticles [13]. These nanoporous gold nanoparticles are expected to broaden the range of applications for both gold nanoparticles and nanoporous gold due to their two-level nanostructures (porosity of around 10 nm and particle size of a few hundreds of nanometers).

Solid-state dewetting of metal films is a simple “bottom-up” approach to fabricate nanoparticles [14–15]. The dewetting of metal films is driven by reducing the surface energy of the film and the interface energy between the film and the substrate, and occurs by diffusion even well below the melting temperature of the film [15]. In addition, alloy nanoparticles can be fabricated by exploiting the dewetting of metallic bilayers [13,16]. By combining both, “top-down” approaches (such as lithography) and “bottom-up” approaches, an ordered array of metallic nanoparticles can be fabricated [15,17–19]. The surface of the substrate is prepatterned into periodic structures by using laser interference lithography [15], focused ion beam (FIB) [17], or substrate conformal imprint lithography (SCIL) [19]. During the dewetting of metal films onto prepatterned substrates, the periodic structure of the prepatterned substrates modulates the local excess chemical potential by the local curvature or by limiting the diffusion paths. This leads to the formation of 2-D nanoparticle arrays with well-defined particle size and particle spacing. Dealloying is a “bottom-up” approach to fabricate nanoporous gold by selectively removing or leaching the element Ag from the Au–Ag alloy in an Ag-corrosive environment [20–22]. In this paper, perfectly ordered arrays of nanoporous gold nanoparticles are fabricated by using a combination of a “top-down” approach (SCIL) and two “bottom-up” approaches (dewetting and dealloying).

## Results and Discussion

**Figure 1:**
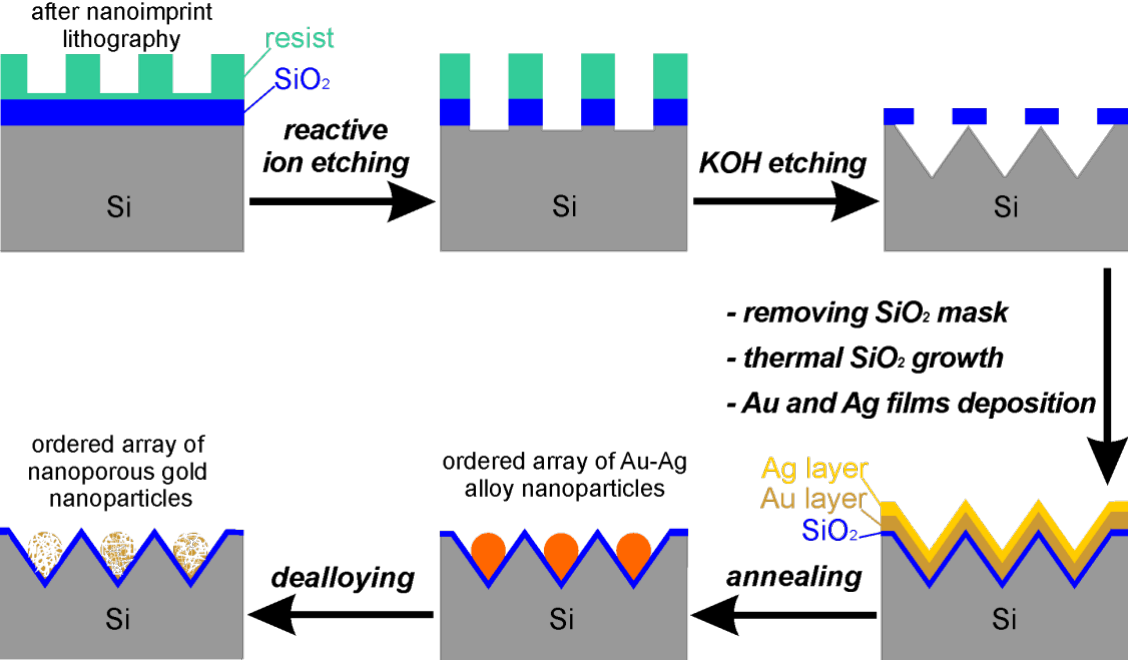
Schematics of the fabrication process for an ordered array of nanoporous gold nanoparticles.

The fabrication process is schematically presented in Figure 1. The surface of a Si(100) wafer was patterned into a periodic array of pyramidal pits (Figure S1, Supporting Information File 1) by using SCIL, reactive ion etching (RIE), and KOH etching. The spatial period of these pits is 520 nm. A 200 nm layer of SiO_2_ was thermally grown on the Si wafer, and then an array of holes was defined by SCIL. SCIL was developed as a new nanoimprint lithography technique that combines the advantages of both UV nanoimprint lithography techniques, with a rigid stamp for best resolution and with a soft stamp for large-area patterning [23]. The soft stamp was carried on glass in SCIL, using a sequential imprinting principle instead of backside pressure to minimize air inclusions on large areas and to ensure high uniformity. The imprinted structure was then transferred through the SiO_2_ layer with RIE. The SiO_2_ pattern with the array of holes acted as a mask during the anisotropic etching of Si in a KOH solution, and the Si surface was patterned into a periodic array of pyramidal pits. After removal of the SiO_2_ mask, about 20 nm of SiO_2_ was then again thermally grown on the structured Si surface to avoid a reaction between the subsequently deposited metal films and the Si substrate. Au/Ag bilayers with different layer thicknesses (10 nm/20 nm, 10 nm/25 nm, 10 nm/30 nm, 15 nm/25 nm, and 15 nm/30 nm) were deposited onto the prepatterned substrates by e-beam evaporation, and then annealed at 700 °C in Ar for 15 min to induce dewetting. This temperature is well below the solidus temperature of the Au–Ag system, i.e., the dewetting is solid-state dewetting. Hence, interdiffusion of Au and Ag occurred, and perfectly ordered arrays of Au–Ag alloy nanoparticles were formed on the prepatterned substrates. Subsequently, dealloying by submerging the samples in a HNO_3_ solution resulted in the transformation of the Au–Ag alloy nanoparticles into the nanoporous gold nanoparticles, due to the dissolution of Ag out of the alloy. As the final result, a well-defined ordered array of nanoporous gold nanoparticles was obtained. The dewetting of the bilayers on the prepatterned substrate with 20 nm thermal SiO_2_ was also performed by annealing at 800 and 900 °C. However, growth of some Si or silicide particles was observed and the particles remained after dealloying; this will be investigated in more detail in the future. In addition, reference samples with 15 nm Au/20 nm Ag bilayers deposited onto a flat Si substrate with 100 nm thick, thermally grown SiO_2_ layer was annealed at 900 °C in Ar for 15 min and then dealloyed for comparison. Dewetting on a prepatterned substrate takes place at a lower annealing temperature [24], and a higher temperature (900 °C) is required for the dewetting on a flat substrate to form particles.

Excess local chemical potential can be introduced by the prepatterned structure according to the Gibbs–Thomson relation, Δμ = κ·γ·Ω, where Δμ is the local excess chemical potential, κ the local curvature, γ the surface energy, and Ω the atomic volume. There is an excess positive chemical potential at peaks or ridges due to the positive local curvature and an excess negative chemical potential at pit valleys due to the negative local curvature. Consequently, there is an additional driving force for the diffusion of the metal atoms from the peaks to the valleys during dewetting on the prepatterned substrate, leading to the formation of the ordered array of nanoparticles. However, in addition to the curvature-driven diffusion, the capillary driven diffusion (the dominating process for the dewetting on a flat substrate [24]) and grain growth are additionally two important processes during dewetting, making the formation of the ordered nanoparticle arrays thickness-dependent [19]. For example, the Au–Ag alloy nanoparticles are irregularly distributed after dewetting 10 nm Au/20 nm Ag bilayers on the prepatterned substrate (Figure S2, Supporting Information File 1). This is probably due to insufficient total layer thickness. As the total layer thickness is increased adequately (Au/Ag: 10 nm/25 nm, 10 nm/30 nm, 15 nm/25 nm, and 15 nm/30 nm), the dewetting on the prepatterned substrate can lead to the formation of ordered arrays of the nanoparticles. It is interesting to note that the optimized thickness for the formation of ordered arrays of particles by dewetting of a thin film in this study is twice as much as that in the previous work [19], although the pit arrays used in this work and the previous work have the same spatial period. However, the pits were fabricated by KOH etching in this study and have a depth of about 360 nm, whereas the pits in the previous work were fabricated by reactive ion etching and have a depth of 150 nm. This means that a larger optimized thickness is required for the formation of an ordered array in the deeper pits with the same spatial period. Additionally, it is possible to control the particle size and spacing by varying the structural parameters (period and depth) of the prepatterned structure, and the layer thickness [19].

**Figure 2:**
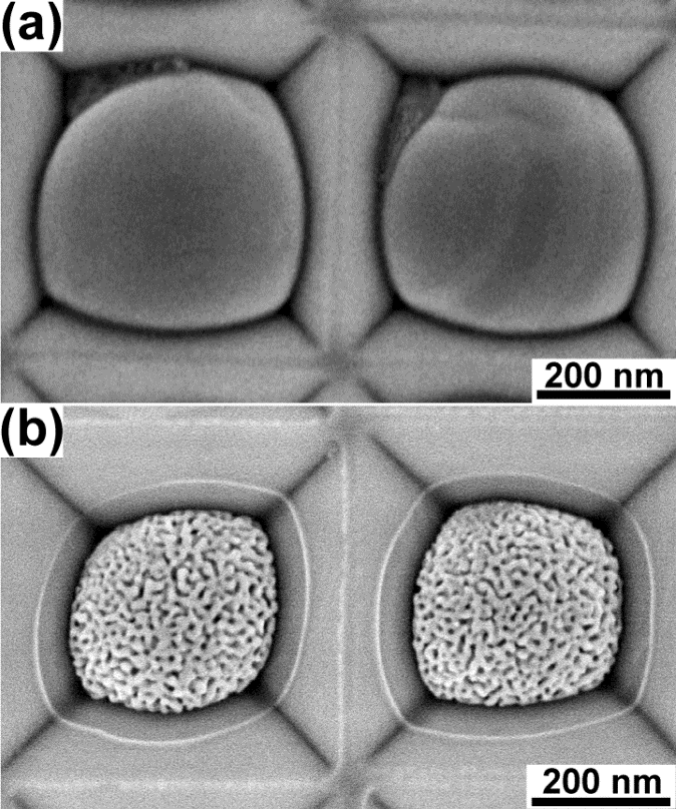
SEM micrographs of samples before and after dealloying: (a) ordered array of Au–Ag alloy nanoparticles dewetted from the 15 nm Au/30 nm Ag bilayers, and (b) ordered array of nanoporous gold nanoparticles formed after the subsequent dealloying.

**Figure 3:**
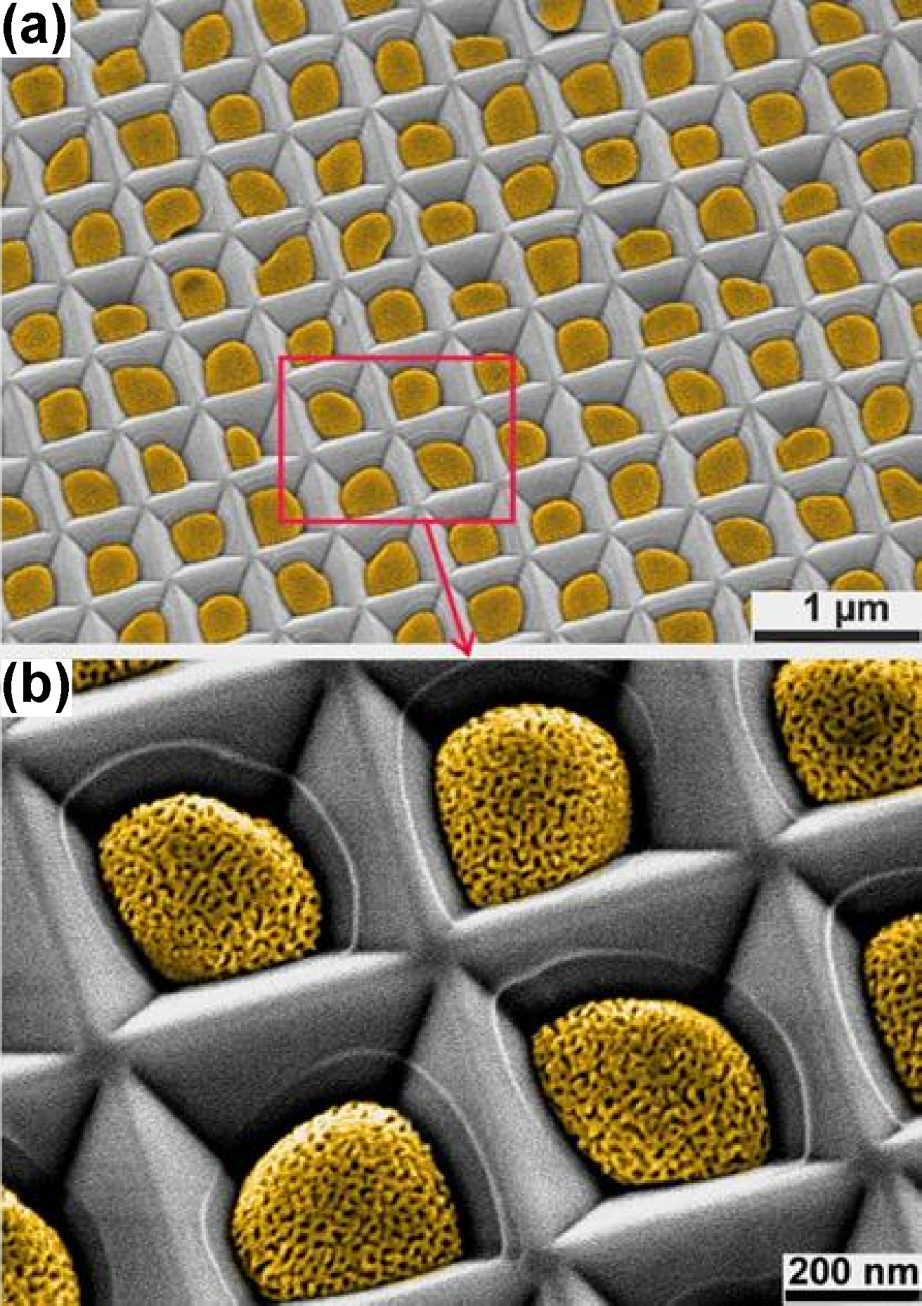
SEM images (false color) at 25° tilt of the perfectly ordered array of the nanoporous gold nanoparticles formed from the 15 nm Au/30 nm Ag bilayers.

Subsequently, dealloying of the ordered arrays of the Au–Ag alloy nanoparticles can result in the formation of ordered arrays of nanoporous gold nanoparticles. Figure 2 shows the SEM images of the ordered array of Au–Ag alloy nanoparticles dewetted from 15 nm Au/30 nm Ag bilayers and the obtained ordered array of nanoporous gold particles after dealloying. The arrays of the nanoporous gold nanoparticles induced from the 10 nm Au/25 nm Ag, 10 nm Au/30 nm Ag, and 15 nm Au/25 nm Ag bilayers are still not perfect, and even instances of two particles in a single pit can be observed (Figure S3, Supporting Information File 1). A perfectly ordered array of nanoporous gold nanoparticles was obtained from the 15 nm Au/30 nm Ag bilayers and there is only one nanoporous gold nanoparticle in every pit. Figure 3a displays the perfectly ordered array of nanoporous gold nanoparticles in a large area, and the porosity of the particles can be seen more clearly in the corresponding magnified SEM image (Figure 3b). In Figure 2b and Figure 3b, the white circular lines outside of the particles can be identified as the previous contours of the dewetted Au–Ag alloy nanoparticles before dealloying. This clearly hints to shrinkage of the particles by dealloying. In a previous work, 29% volume shrinkage of the nanoparticles was reported after dealloying [13].

**Figure 4:**
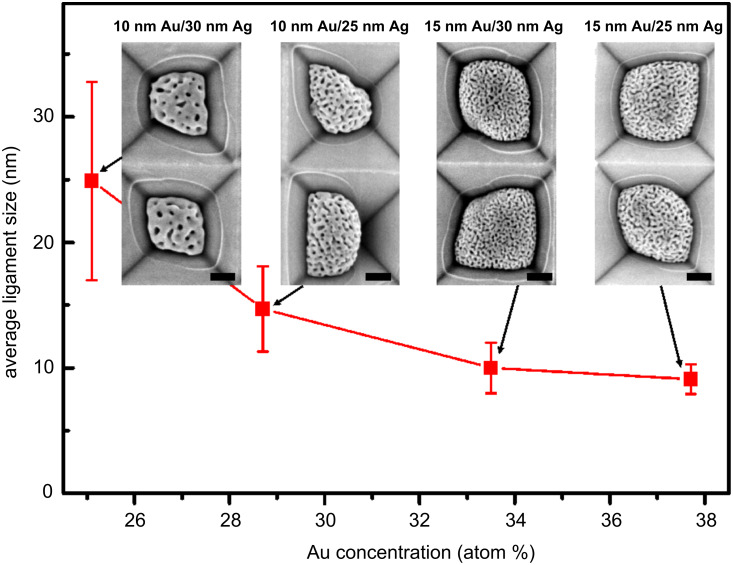
Plot of the average ligament size as a function of the Au concentration of alloy nanoparticles. The error bars represent the standard deviation. Insets show the corresponding SEM images. Scale bars in the insets are 100 nm.

The surface morphology of the nanoporous gold particles on the prepatterned substrates changes with the thickness ratio of the as-deposited bilayers. Au and Ag are fully miscible, thus the Au concentration in the formed Au–Ag alloy nanoparticles can be roughly calculated based on the layer thickness ratio. It is 29 atom % for the 10 nm Au/25 nm Ag bilayers, 25 atom % for the 10 nm Au/30 nm Ag bilayers, 38 atom % for the 15 nm Au/25 nm Ag bilayers, and 34 atom % for the 15 nm Au/30 nm Ag bilayers. The average ligament size *<*θ*>* is plotted as a function of the Au concentration, as shown in Figure 4. *<*θ*>* decreases from 25 nm (for the particles induced from the 10 nm Au/30 nm Ag bilayers) to 9 nm (for the particles induced from the 15 nm Au/25 nm Ag bilayers) with increasing Au concentration. As Au concentration approaches above 34 atom %, the ligament size seems to approach a lower saturation value of *<*θ*>* = 10 nm. Therefore, the ligament size (or pore size) of the nanoporous nanoparticles can be controlled by varying the layer-thickness ratio of the as-deposited Au/Ag bilayers. The size shrinkage decreases with increasing Au concentration, as observed from the contours (white circular lines) of the original Au–Ag alloy nanoparticles and those of the nanoporous gold nanoparticles (inset SEM images in Figure 4).

**Figure 5:**
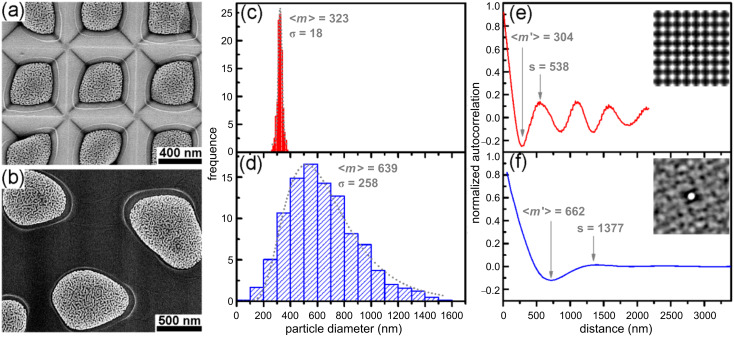
Data for the ordered array of nanoporous gold nanoparticles formed from the 15 nm Au/30 nm Ag multilayers on the prepatterned substrate (a, c, and e) and the irregularly distributed nanoporous gold nanoparticles formed from the 15 nm Au/20 nm Ag bilayers on the flat substrate (b, d, and f). (a, b) SEM images, (c, d) histograms of the particle diameter distributions, and (e, f) plots of radially averaged autocorrelation. Fitting curves (log-normal function) are superimposed on the histograms. The values <m> or <m′> and σ indicate the mean particle diameter and its standard deviation, and “s” denotes the characteristic particle spacing (all in nm). Insets in (e) and (f) show the corresponding autocorrelation images.

Figure 5 shows a comparison of the data from both the array of nanoporous gold nanoparticles induced from the 15 nm Au/30 nm Ag bilayers on the prepatterned substrate and the irregularly distributed nanoporous gold nanoparticles induced from the 15 nm Au/20 nm Ag bilayers on the flat substrate. Figure 5a and Figure 5b are the SEM images, Figure 5c and Figure 5d are histograms of the particle size distribution, and the mean particle size <*m*> is determined. Figure 5e and Figure 5f are the plots of the radially averaged autocorrelation. The radially averaged autocorrelation is calculated from the autocorrelation or pair correlation as a function of radial distance. The first minimum of this function shows information about the mean particle size <*m*′>, and the subsequent first maximum denotes the characteristic particle spacing *s*. There is a small difference between <*m*> and <*m*′> due to the different determination methods. Normally, the mean particle size or diameter <*m*>, width of the particle size distribution <λ_p_>, and the characteristic particle spacing *s* increase with increasing film thickness for the dewetted nanoparticles on flat substrates [19]. However, the prepatterned substrates with nanostructures lead to an obvious reduction of the particle size and spacing [19]. Although the total bilayer thickness of the as-deposited bilayers for the ordered array of nanoporous gold nanoparticles is clearly larger than that for the nanoporous gold nanoparticles on the flat substrate, it can be seen that the mean particle diameter (323 nm) and characteristic particle spacing (538 nm) of the ordered array of nanoporous gold nanoparticles are much smaller than those (639 nm and 1377 nm) of the nanoporous gold nanoparticles induced on the flat substrate. The particle size distribution for the array of nanoporous gold nanoparticles on the prepatterned substrate possesses a much smaller width comparing to that for the nanoporous gold nanoparticles induced on the flat substrate, i.e., the particles on the prepatterned substrate are much more uniform and almost perfectly ordered. The characteristic particle spacing (538 nm) of the array of the nanoporous gold nanoparticles corresponds nearly to the spatial period (520 nm) of the pits of the prepatterned substrate, and the small deviation is probably due to the uncertainty of the radially averaged autocorrelation. Comparing the radially averaged autocorrelation of the nanoporous gold nanoparticles on the flat substrate, the plot of the array of the nanoporous gold nanoparticles on the prepatterned substrate shows a periodic wave shape, denoting the high regularity of the nanoparticle array, which is well confirmed by the autocorrelation image (inset in Figure 5e).

## Conclusion

In summary, a combination of a “top-down” approach and “bottom-up” approaches is used to fabricate perfectly ordered arrays of nanoporous gold nanoparticles, which cannot be produced by using “top-down” or “bottom-up” techniques alone. By using the SCIL technique, large surface areas can be prepatterned into uniform periodic nanostructures, and correspondingly, large areas of well-ordered arrays of nanoporous gold nanoparticles can be fabricated. In addition, it is possible to control the characteristics of both particles (particle size and spacing) and porosity (ligament size) by varying the structural parameters of the prepatterned structure, total layer thickness, and the layer-thickness ratio of the as-deposited Au/Ag bilayers. This regular arrangement of nanoporous gold nanoparticles with three-level nanostructures (ligament size of tens of nanometers, particle size of a few hundred nanometers, and well-defined particle size and spacing) is expected to broaden the application areas of both the nanoparticles and nanoporous materials.

## Experimental

The surface of a Si(100) wafer was structured into periodic array of pyramidal pits by using SCIL, reactive ion etching (RIE, Oxford Plasmalab 100), and KOH etching. Before application of the resist for SCIL, 200 nm of SiO_2_ was thermally grown on the Si wafer. A pattern with an array of holes was defined by SCIL, and then transferred to the SiO_2_ layer by RIE. The SiO_2_ pattern with the array of holes acted as a mask during the anisotropic etching of Si in a 40 wt % KOH solution at 60 °C, and a periodic array of pyramidal pits was formed. Then, the SiO_2_ mask was removed by using a 7 wt % HF solution. About 20 nm of SiO_2_ was then thermally grown. Au/Ag bilayers were deposited on the substrates by e-beam evaporation and then annealed at 700 °C in Ar for 15 min to induce dewetting. Then, dealloying was performed by submerging the samples in a 65 wt % HNO_3_ solution at 21 °C for 5 min. A reference sample (15 nm Au/20 nm Ag bilayers on a flat SiO_2_/Si substrate) was processed by annealing at 900 °C in Ar for 15 min and then submerging in a 65 wt % HNO_3_ solution at 21 °C for 5 min. The SiO_2_ thickness of the reference sample is 100 nm. The samples were investigated using an ultra-high-resolution scanning electron microscope (FE-SEM, Hitachi S-4800). Particle diameters were recalculated as circular diameters and measured by thresholding the image contrast in the SEM images and counting pixels. The average ligament size, which is defined as the equivalent diameter of ligaments in the nanoporous gold nanoparticles, was determined manually by identifying a minimum of 20 ligaments, measuring across the shortest distance of each ligament, and then averaging. The radially averaged autocorrelation is calculated from the autocorrelation (also known as pair correlation) of pixels of a converted binary image as a function of their radial distance. In the binary images, the areas of particles and background can be best identified.

## Supporting Information

File 1 Additional SEM images.
