# Supplementary material for: Ordered arrays of nanoporous gold nanoparticles
Source: Beilstein J Nanotechnol. 2012 Sep 13;3:651–7. doi: 10.3762/bjnano.3.74 (PMC3458611; doi:10.3762/bjnano.3.74)
Supplement: File 1 — Additional SEM images. [file Beilstein_J_Nanotechnol-03-651-s001.pdf]

Supporting Information

for

Ordered arrays of nanoporous gold nanoparticles

Dong Wang*[1,§], Ran Ji[2], Arne Albrecht[3] and Peter Schaaf*[1,¶]

Address: [1]Chair Materials for Electronics, Institute of Materials Engineering and Institute of Micro- and Nanotechnologies MacroNano®, Ilmenau University of Technology, POB 10 05 65, 98684 Ilmenau, Germany, [2]SÜSS MicroTec Lithography GmbH, Schleissheimer Str. 90, 85748 Garching, Germany and [3]Center for Micro- and Nanotechnologies, Ilmenau University of Technology, POB 10 05 65, 98684 Ilmenau, Germany

Email: Dong Wang* - dong.wang@tu-ilmenau.de;
Peter Schaaf* - peter.schaaf@tu-ilmenau.de

*Corresponding author

[§]Tel.: +49 3677 69 3170, Fax: + 3677 69 3171;

[¶]Tel.: +49 3677 69 3611, Fax: + 3677 69 3171

Additional SEM images

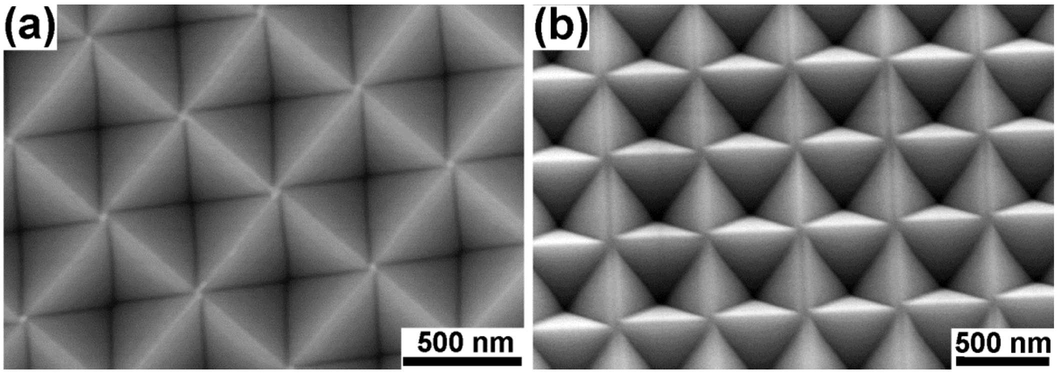

Figure S1: SEM images of the pre-patterned substrate showing the periodic array of pyramidal pits: (a) plan view, and (b) at tilt of 25°.

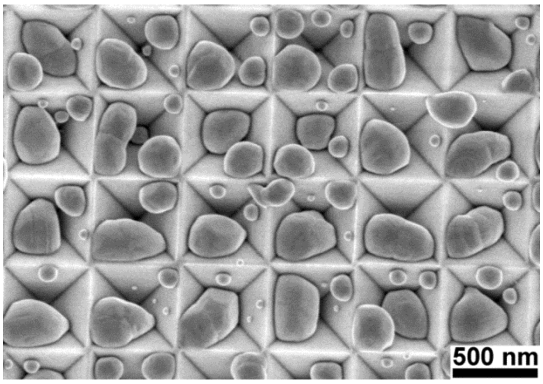

Figure S2: SEM image of the Au–Ag alloy nanoparticles dewetted from 10 nm Au/20 nm Ag bilayers on the pre-patterned substrate via annealing at 700 °C in Ar for 15 min.

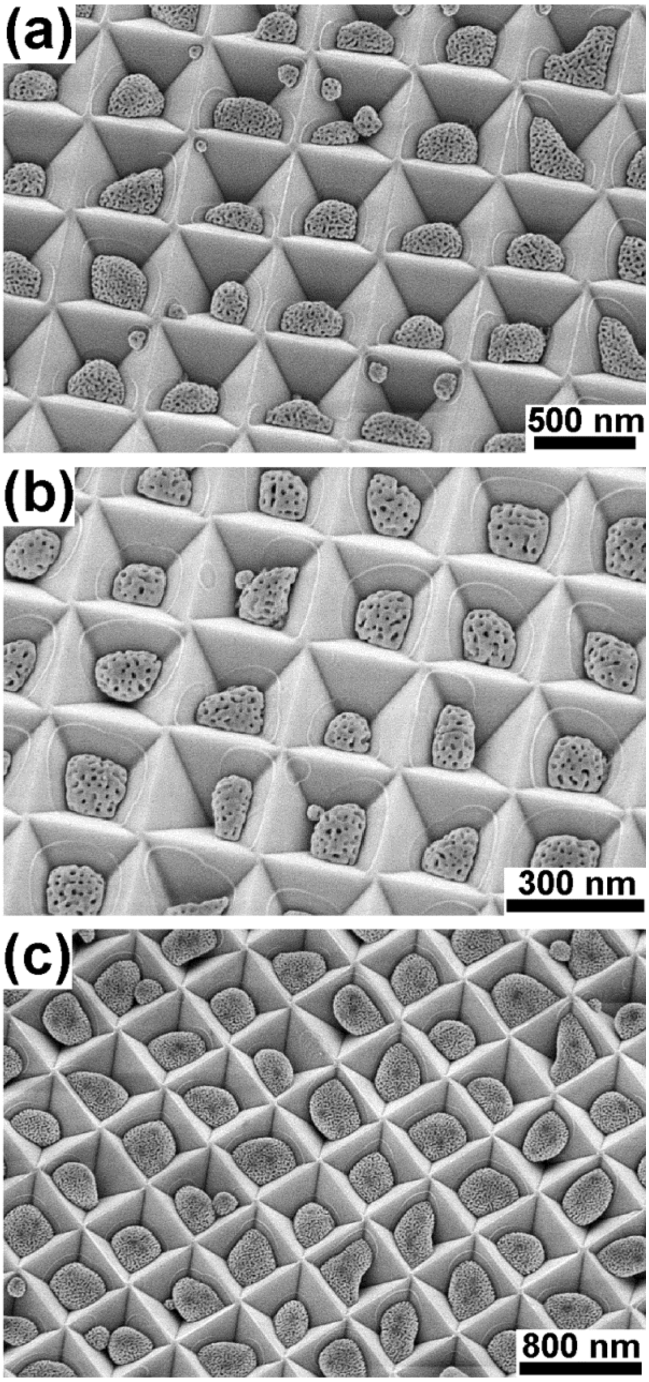

Figure S3: SEM images (at tilt of 25°) of arrays of nanoporous gold nanoparticles induced via annealing at 700 °C in Ar for 15 min and then dealloying from: (a) 10 nm Au/25 nm Ag bilayers, (b) 10 nm Au/30 nm Ag bilayers, and (c) 15 nm Au/25 nm Ag bilayers.
